# Supplementary material for: Alterations in Gut Microbiota After Upper Gastrointestinal Resections: Should We Implement Screening to Prevent Complications?
Source: Medicina (Kaunas). 2025 Oct 11;61(10):1822. doi: 10.3390/medicina61101822 (PMC12566039; doi:10.3390/medicina61101822)
Supplement: Supplementary file 1 [file medicina-61-01822-s001.zip › medicina-3873311-supplementary.pdf]

Supplementary Material S1. Questionnaire used for assessment of gastrointestinal symptoms

Part 1: Demographic and Anthropometric Data

Participant code/name: ___________________________

Date of survey completion: ________________________

Gender: ☐ M ☐ F

Body weight: _________ kg Age: _________ years Body height: _________ cm

1. Level of education (please tick one):
☐ Incomplete primary school
☐ Primary school
☐ Lower vocational education (2 years)
☐ Higher vocational education (3 years)
☐ General secondary school / technical / other secondary education
☐ VI or higher

2. Socioeconomic status:

Imagine a ladder with 10 steps. Which step would you place yourself on according to your socioeconomic status in society?

(Lower steps = lower socioeconomic status)

Step: ____

3. Time since surgery:

☐ 3 months ☐ 6 months ☐ 9 months or more

4. Postoperative course:

☐ Without complications ☐ With complications

5. Past medical history (please tick all that apply):

☐ Stomach cancer

☐ Pancreatic cancer

☐ Morbid obesity

6. Surgical history (due to the above conditions):

☐ Gastrectomy (stomach removal)

☐ Pancreatectomy (pancreas removal)

☐ Bariatric surgery (e.g. gastric bypass)

☐ Intestinal resection due to Crohn's disease

7. Treatment before surgery:

☐ Chemotherapy ☐ Radiation therapy ☐ Other: ____________ ☐ None

8. Treatment after surgery:

☐ Chemotherapy ☐ Radiation therapy ☐ Other: ____________ ☐ None

9. Antibiotic treatment after surgery:

Name of antibiotic: ____________________

Duration of treatment: __________________

10. In the past few months have you taken any of the following:

☐ Vitamin B12 ☐ Iron

Part 2: Symptoms (Gastrointestinal Symptom Rating Scale)

Do you have any of the following symptoms? Please rate the severity on a scale from 1 to 10, where 1 = no symptoms and 10 = very severe symptoms.

No.	Question.	Scale.
1.	Chronic abdominal pain (if yes, where: around the navel. upper right, upper left, lower right, lower left)	1 2 3 4 5 6 7 8 9 10
2.	Diarrhea (frequency: 3+ daily, 1-2 daily, 5-6 weekly, 3-4 weekly, <3 weekly)	1 2 3 4 5 6 7 8 9 10
3.	Constipation	1 2 3 4 5 6 7 8 9 10
4.	Floating stools / multiple flushing needed (YES / NO)	1 2 3 4 5 6 7 8 9 10
5.	Abdominal cramps	1 2 3 4 5 6 7 8 9 10
6.	Bloating and flatulence	1 2 3 4 5 6 7 8 9 10
7.	Nausea	1 2 3 4 5 6 7 8 9 10
8.	Vomiting	1 2 3 4 5 6 7 8 9 10
9.	Acid reflux / heartburn	1 2 3 4 5 6 7 8 9 10
10.	Loss of appetite	1 2 3 4 5 6 7 8 9 10
11.	Feeling bloated after meals	1 2 3 4 5 6 7 8 9 10
12.	Fever	1 2 3 4 5 6 7 8 9 10
13.	Joint pain	1 2 3 4 5 6 7 8 9 10
14.	Fatigue	1 2 3 4 5 6 7 8 9 10
15.	Skin problems (rosacea/rashes/eczema)	1 2 3 4 5 6 7 8 9 10
16.	Weight changes in last 3 months (lost/gained/no change): ______ kg	1 2 3 4 5 6 7 8 9 10

17.	Brain fog	1 2 3 4 5 6 7 8 9 10
18.	Mood changes	1 2 3 4 5 6 7 8 9 10

Part 3. SIBO Questionnaire

Please answer each question according to how much it applies to you, using a scale from 0 (not at all/never) to 4 (very much/always).

Question	Scale
I have bloating/gas.	0 1 2 3 4
I burp after meals.	0 1 2 3 4
I have abdominal pain and bloating.	0 1 2 3 4
I have constipation.	0 1 2 3 4
Constipation worsens when I eat fiber (e.g. legumes).	0 1 2 3 4
My symptoms alternate between constipation and diarrhea.	0 1 2 3 4
I developed gut issues after taking opioids.	0 1 2 3 4
I have diarrhea.	0 1 2 3 4
I have both constipation and diarrhea.	0 1 2 3 4
I have gastroesophageal reflux disease/heartburn.	0 1 2 3 4
I have nausea or nausea with burping.	0 1 2 3 4
I have increased intestinal permeability.	0 1 2 3 4
I have irritable bowel syndrome (IBS).	0 1 2 3 4
I have Crohn's disease or ulcerative colitis.	0 1 2 3 4
I have diverticulitis.	0 1 2 3 4
I have food intolerances.	0 1 2 3 4
I have lactose intolerance.	0 1 2 3 4
I have difficulty consuming milk.	0 1 2 3 4
I have celiac disease or gluten sensitivity.	0 1 2 3 4
I have celiac disease. follow a gluten-free diet but still feel unwell.	0 1 2 3 4
I have gluten sensitivity. follow a gluten-free diet but still feel unwell.	0 1 2 3 4
My pancreas was not visible on CT due to air bubbles.	0 1 2 3 4
I have fatty stools (steatorrhea).	0 1 2 3 4
I have non-alcoholic fatty liver disease.	0 1 2 3 4
I have liver cirrhosis.	0 1 2 3 4
I have fibromyalgia.	0 1 2 3 4
I have chronic fatigue syndrome.	0 1 2 3 4
I have joint pain.	0 1 2 3 4
I have restless legs syndrome.	0 1 2 3 4
I have skin problems: eczema, atopic dermatitis, or psoriasis.	0 1 2 3 4
I have rosacea or acne rosacea.	0 1 2 3 4
I have scleroderma or lupus.	0 1 2 3 4
I have breathing difficulties.	0 1 2 3 4
I have headaches.	0 1 2 3 4
I have memory problems.	0 1 2 3 4
I have autism.	0 1 2 3 4
I have anemia related to vitamin B12 deficiency.	0 1 2 3 4
I have anemia related to iron deficiency.	0 1 2 3 4
I have low ferritin levels without a clear cause.	0 1 2 3 4
I have chronic vitamin D deficiency.	0 1 2 3 4
I have interstitial cystitis.	0 1 2 3 4
I have type 1 or type 2 diabetes.	0 1 2 3 4

I have hypothyroidism.	0 1 2 3 4
My gut symptoms improve significantly after antibiotics.	0 1 2 3 4
I cannot tolerate probiotics. or my symptoms worsen with probiotics/prebiotics.	0 1 2 3 4
My symptoms worsen when I eat fiber.	0 1 2 3 4
Gut symptoms developed after food poisoning or travel.	0 1 2 3 4
I prefer snacking to regular meals.	0 1 2 3 4
I like eating starch/grains/carbohydrates and often include them in my meals.	0 1 2 3 4
I currently eat or have eaten a lot of starch/grains/carbohydrates and feel unwell after eating them.	0 1 2 3 4
I used to eat a lot of starch/grains/carbohydrates but no longer do.	0 1 2 3 4
I was born in the C-section.	0 1 2 3 4
I was not breastfed.	0 1 2 3 4
My mother (or grandmother) has similar gut problems.	0 1 2 3 4
As a child, I often ate alone or in front of the TV rather than at the table.	0 1 2 3 4
As a child, I often received antibiotics (e.g. for ear infections. tonsillitis).	0 1 2 3 4
As an adult, I regularly or frequently received antibiotics.	0 1 2 3 4
As an adult, I regularly receive oral contraceptives.	0 1 2 3 4
I experienced a period of severe stress or trauma.	0 1 2 3 4
I have taken morphine or opioids.	0 1 2 3 4

Part 4. 36-Item Short Form Survey Instrument (SF-36).

Available at: https://www.rand.org/health-care/surveys_tools/mos/36-item-short-form/survey-instrument.html

Supplementary Material S2. Gastrointestinal symptoms according to SIBO status stratified by surgical subgroup after upper gastrointestinal resections

Supplementary Table S1 Gastrointestinal symptoms according to SIBO status stratified by surgical subgroup after upper gastrointestinal resections.

	RYGBP/OAGBP			STG in TG			SP in WR/TP			CD		
	SIBO + (n = 24)	SIBO - (n = 32)	*p*-value	SIBO + (n = 11)	SIBO - (n = 26)	*p*-value	SIBO + (n = 8)	SIBO - (n = 30)	*p*-value	SIBO + (n = 5)	SIBO - (n =21)	*p*-value
CP [n] (%)	2.38 ± 1.88	2.25 ± 1.85	0.907	2.27 ± 1.85	2.27 ± 2.38	0.795	2.25 ± 2.55	1.93 ± 1.55	0.845	2.80 ± 1.48	3.33 ± 2.08	0.691
Diarrhoea	2.96 ± 2.73	3.03 ± 2.21	0.387	1.82 ± 1.33	2.42 ± 2.04	0.435	3.63 ± 3.54	2.00 ± 2.17	0.175	6.60 ± 1.95	5.24 ± 2.72	0.219
Frequency of defecation	2.58 ± 1.10	2.03 ± 1.00	0.022*	2.18 ± 0.60	1.98 ± 0.65	0.320	1.63 ± 0.52	2.25 ± 0.84	0.046*	1.00 ± 0.00	1.81 ± 1.12	0.061
Constipation	2.58 ± 2.45	1.78 ± 1.54	0.356	2.00 ± 2.72	1.54 ± 1.17	0.853	1.25 ± 0.71	2.00 ± 2.10	0.320	1.20 ± 0.45	1.95 ± 2.20	0.616
Floating stools	1.54 ± 0.51	1.56 ± 0.50	0.878	1.45 ± 0.52	1.77 ± 0.43	0.065	1.63 ± 0.52	1.77 ± 0.43	0.425	1.80 ± 0.45	1.76 ± 0.44	0.859
Abdominal crams	2.96 ± 2.35	2.53 ± 1.85	0.739	2.23 ± 1.51	2.27 ± 2.38	0.580	2.25 ± 1.75	1.90 ± 1.35	0.569	2.60 ± 1.52	3.81 ± 2.50	0.356
Bloating and flatulence	5.71 ± 3.33	4.25 ± 2.45	0.104	6.18 ± 3.40	4.42 ± 2.67	0.107	6.38 ± 2.00	3.97 ± 2.99	0.030*	5.00 ± 3.16	4.62 ± 2.94	0.792
Nausea	1.67 ± 1.40	2.00 ± 2.08	0.596	2.00 ± 1.79	1.85 ± 1.74	0.447	1.00 ± 0.00	1.50 ± 1.36	0.282	1.60 ± 0.89	1.81 ± 1.57	0.739
Vomiting	1.46 ± 1.06	1.38 ± 1.29	0.585	1.00 ± 0.00	1.77 ± 1.68	0.087	1.00 ± 0.00	1.00 ± 0.26	1.00	1.00 ± 0.00	1.24 ± 0.89	0.482
Belching	2.38 ± 1.79	3.28 ± 2.74	0.357	4.00 ± 3.35	2.65 ± 2.23	0.413	2.25 ± 2.31	2.33 ± 2.41	0.671	2.20 ± 2.17	2.38 ± 2.29	0.908
Appetite loss	1.50 ± 1.14	1.34 ± 1.00	0.429	1.91 ± 2.39	2.31 ± 2.59	0.773	1.13 ± 0.35	1.43 ± 1.10	0.578	1.80 ± 1.30	1.43 ± 1.21	0.256
Bloating	3.96 ± 2.84	3.34 ± 2.27	0.503	4.27 ± 3.26	3.46 ± 2.55	0.465	3.00 ± 2.39	2.87 ± 2.86	0.454	4.00 ± 2.55	4.19 ± 3.12	0.894
Fever	1.17 ± 0.82	1.06 ± 0.25	0.766	1.00 ± 0.00	1.00 ± 0.00	NA	1.13 ± 0.35	1.13 ± 0.73	0.331	1.00 ± 0.00	1.00 ± 0.00	NA
Joint pain	2.79 ± 2.08	2.81 ± 2.53	0.765	1.82 ± 1.83	2.69 ± 2.19	0.162	1.50 ± 1.07	2.43 ± 2.22	0.341	3.20 ± 0.84	3.86 ± 2.50	0.765
Fatigue	3.63 ± 2.63	3.19 ± 2.69	0.461	3.05 ± 3.10	3.54 ± 2.97	0.605	3.50 ± 1.69	3.10 ± 2.72	0.301	6.20 ± 3.27	4.57 ± 2.38	0.308
Skin manifestations	2.88 ± 2.83	1.59 ± 1.60	0.068	1.64 ± 1.80	1.57 ± 1.45	0.942	1.38 ± 0.52	1.73 ± 1.60	0.660	1.40 ± 0.89	2.14 ± 2.15	0.466
Altered mental status	1.88 ± 1.60	2.00 ± 2.09	0.670	1.73 ± 1.35	2.04 ± 2.01	0.789	2.63 ± 1.92	1.73 ± 1.53	0.053	1.80 ± 1.30	2.33 ± 1.88	0.690
Flatulence	3.00 ± 1.06	2.63 ± 0.83	0.135	2.5 ± 1.6	2.3 ± 1.0	0.524	3.50 ± 0.76	2.40 ± 1.19	0.019*	2.00 ± 1.41	2.62 ± 1.20	0.287
Belching after meals	1.83 ± 1.34	1.38 ± 1.16	0.205	1.91 ± 1.51	2.04 ± 1.48	0.786	1.25 ± 1.17	1.07 ± 1.23	0.636	1.00 ± 1.22	1.23 ± 1.09	0.636
Abdominal pain and bloating	1.54 ± 1.22	1.53 ± 1.08	1.000	1.18 ± 1.54	1.40 ± 1.34	0.490	1.25 ± 1.49	1.20 ± 1.27	1.000	1.60 ± 1.34	1.86 ± 1.28	0.736
CD	0.92 ± 1.25	0.97 ± 1.15	0.756	0.36 ± 0.92	0.31 ± 0.68	0.817	0.25 ± 0.71	0.50 ± 1.07	0.456	1.40 ± 0.89	1.00 ± 1.30	0.377

Constipation and diarrhoea	1.00 ± 1.41	0.75 ± 0.92	0.835	0.27 ± 0.65	0.31 ± 0.55	0.657	0.00 ± 0.00	0.47 ± 1.11	0.176	1.00 ± 1.00	1.00 ± 1.30	0.830

Values are presented as mean ± standard deviation (SD) or number (percentage). Statistical analysis was performed using the χ^2 test, Fisher's exact test, Mann–Whitney U test, and Student's *t*-test, as appropriate. CD – alternating constipation and diarrhoea; CP – chronic pain.
$p < 0.05$ indicates statistical significance.
